# Validation of the age-adjusted shock index for pediatric casualties in Iraq and Afghanistan

**DOI:** 10.1186/s40779-020-00262-8

**Published:** 2020-07-02

**Authors:** Camaren M. Cuenca, Matthew A. Borgman, Michael D. April, Andrew D. Fisher, Steven G. Schauer

**Affiliations:** 1 US Army Institute of Surgical Research, 3698 Chambers Pass, JBSA Fort Sam Houston, San Antonio, TX 78234-7767 USA; 2 Brooke Army Medical Center, JBSA Fort Sam Houston, San Antonio, TX USA; 3 Uniformed Services University of the Heath Sciences, Bethesda, MD USA; 4 Texas Army National Guard, Austin, TX USA; 5 Department of Surgery, UNM School of Medicine, Albuquerque, NM USA; 6 59th Medical Wing, JBSA Lackland, San Antonio, TX USA

**Keywords:** Pediatric, Massive, Transfusion, Shock, Index, Age

## Abstract

**Background:**

Pediatric casualties account for a notable proportion of encounters in the deployed setting based on the humanitarian medical care mission. Previously published data demonstrates that an age-adjust shock index may be a useful tool in predicting massive transfusion and death in children. We seek to determine if those previous findings are applicable to the deployed, combat trauma setting.

**Methods:**

We queried the Department of Defense Trauma Registry (DODTR) for all pediatric subjects admitted to US and Coalition fixed-facility hospitals in Iraq and Afghanistan from January 2007 to January 2016. This is a secondary analysis of casualties seeking to validate previously published data using the shock index, pediatric age-adjusted. We then used previously published thresholds to determine patients outcome for validation by age grouping, 1–3 years (1.2), 4–6 years (1.2), 7–12 years (1.0), 13–17 years (0.9).

**Results:**

From January 2007 through January 2016 there were 3439 pediatric casualties of which 3145 had a documented heart rate and systolic pressure. Of those 502 (16.0%) underwent massive transfusion and 226 (7.2%) died prior to hospital discharge. Receiver operating characteristic (ROC) thresholds were inconsistent across age groups ranging from 1.0 to 1.9 with generally limited area under the curve (AUC) values for both massive transfusion and death prediction characteristics. Using the previously defined thresholds for validation, we report sensitivity and specificity for the massive transfusion by age-group: 1–3 (0.73, 0.35), 4–6 (0.63, 0.60), 7–12 (0.80, 0.57), 13–17 (0.77, 0.62). For death, 1–3 (0.75, 0.34), 4–6 (0.66–0.59), 7–12 (0.64, 0.52), 13–17 (0.70, 0.57). However, negative predictive values (NPV) were generally high with all greater than 0.87.

**Conclusions:**

Within the combat setting, the age-adjusted pediatric shock index had moderate sensitivity and relatively poor specificity for predicting massive transfusion and death. Better scoring systems are needed to predict resource needs prior to arrival, that perhaps include other physiologic metrics. We were unable to validate the previously published findings within the combat trauma population.

## Background

Traumatic injury is the leading cause of death in children and adolescents [1, 2]. Pediatric patients with traumatic injuries are frequently seen at US military medical facilities due to both combat and non-combat related incidents based on the humanitarian mission requirements. When compared to adults in the deployed combat setting, the pediatric population has an increased mortality for similar injuries [3]. This is due to a multitude of factors, including increased severity of injury and increased rates of coagulopathy, shock, and acidosis upon admission [4–6]. Children comprise a significant portion of traumatic injuries that occur in a modern war zone, emphasizing the need for military medical facilities to be adequately prepared for a high volume of pediatric casualties requiring care [5, 7].

Activation of trauma care resources prior to patients arrival is known to decrease mortality in severely injured children reducing the time to life-saving interventions, such as a blood products [8]. Multiple scoring systems have been developed to categorize traumatically injured patients in order to guide triage and care management. Physiologic scoring systems can provide a real-time evaluation of the degree to which the patient has been affected by trauma using physiologic parameters and exam findings [9]. Such examples include the shock index, which is patient’s heart rate divided by the systolic blood pressure. In adults, an SI above 0.9 at time of hospital arrival is associated with increased mortality and need for massive transfusion [10–12]. However, this system was developed for use on adults, and as with many systems, was secondarily applied to pediatric patients [13].

A study by Acker et al. [14] modified the SI 0.9 cut-off value using age-based vital sign parameters to become the shock index, pediatric-age adjusted (SIPA). They reported that SIPA more accurately predicted pediatric patient outcomes, including injury severity, intensive care unit (ICU) admission, transfusion requirements, and mortality. Nordin et al. [15] conducted a study that validated the SIPA, determining that it was a better predictor of pediatric patient outcomes with a higher specificity and positive predictive value than SI. While this study provided a valuable validation of the SIPA and its thresholds, it was conducted with data solely from the civilian setting. No study has yet to analyze SIPA value for pediatric patients in the combat setting which carries a higher frequency of gunshot wounds and explosive polytrauma. The ability to predict which pediatric casualties will require resuscitation allows for preparation of resources, especially when activation of the walking blood bank. Moreover, with significant variability in transport times, the US military has developed methods to bring blood to the point-of-injury, which would help inform the transport teams. So, we are seeking to validate the previously published data by Nordin et al. [15] in a combat trauma population.

## Methods

### Subjects and setting

This is a secondary analysis of a previously published dataset with a focus on developing a risk prediction model for pediatric casualties that will undergo massive transfusion [16, 17]. Our methods for identifying massive transfusion in pediatric casualties are previously described [18]. The US Army Institute of Surgical Research regulatory office reviewed protocol H-16-014 and determined it was exempt from institutional review board oversight. We obtained only de-identified data.

### Department of Defense Trauma Registry (DODTR) description

We queried the Department of Defense Trauma Registry (DODTR) for all pediatric (age < 18 years) encounters from January 2007 to January 2016. The DODTR, formerly known as the Joint Theater Trauma Registry (JTTR), is the data repository for DoD trauma-related injuries [16, 17, 19–22]. The DODTR includes documentation regarding demographics, injury-producing incidents, diagnoses, treatments, and outcomes of injuries sustained by US/non-US military and US/non-US civilian personnel in wartime and peacetime from the point of injury to final disposition. The DODTR comprises all patients admitted to a Role 3 (fixed-facility) or forward surgical team (FST) with an injury diagnosis using the International Classification of Disease 9th Edition (ICD-9) between 800.0–959.9, near-drowning/drowning with associated injury (ICD-9994.1) or inhalational injury (ICD-9987.9) and trauma occurring within 72 h from presentation. This study comprises a retrospective review of prospectively collected data within the registry. We requested all available documentation of prehospital care and fixed-facility based care.

### Statistical analysis

We performed all statistical analysis using Microsoft Excel (version 10, Redmond, Washington) and JMP Statistical Discovery from SAS (version 13, Cary, NC). We compared study variables using a student *t*-test for continuous variables, Wilcoxon Rank Sum test for ordinal variables, and chi-squared test for nominal variables. We used nominal logistic regression analyses for receiver operating characteristic (ROC) thresholds and analyses with areas under the curve (AUC) for model fit. We then used previously published thresholds for validation by age grouping, 1–3 years (1.2), 4–6 years (1.2), 7–12 years (1.0), 13–17 years (0.9) [15]. Emergency department vitals were used as a surrogate due to limitations in prehospital documentation [23]. Specifically, we used the highest documented heart rate and the lowest document systolic pressure.

## Results

**Fig. 1:**
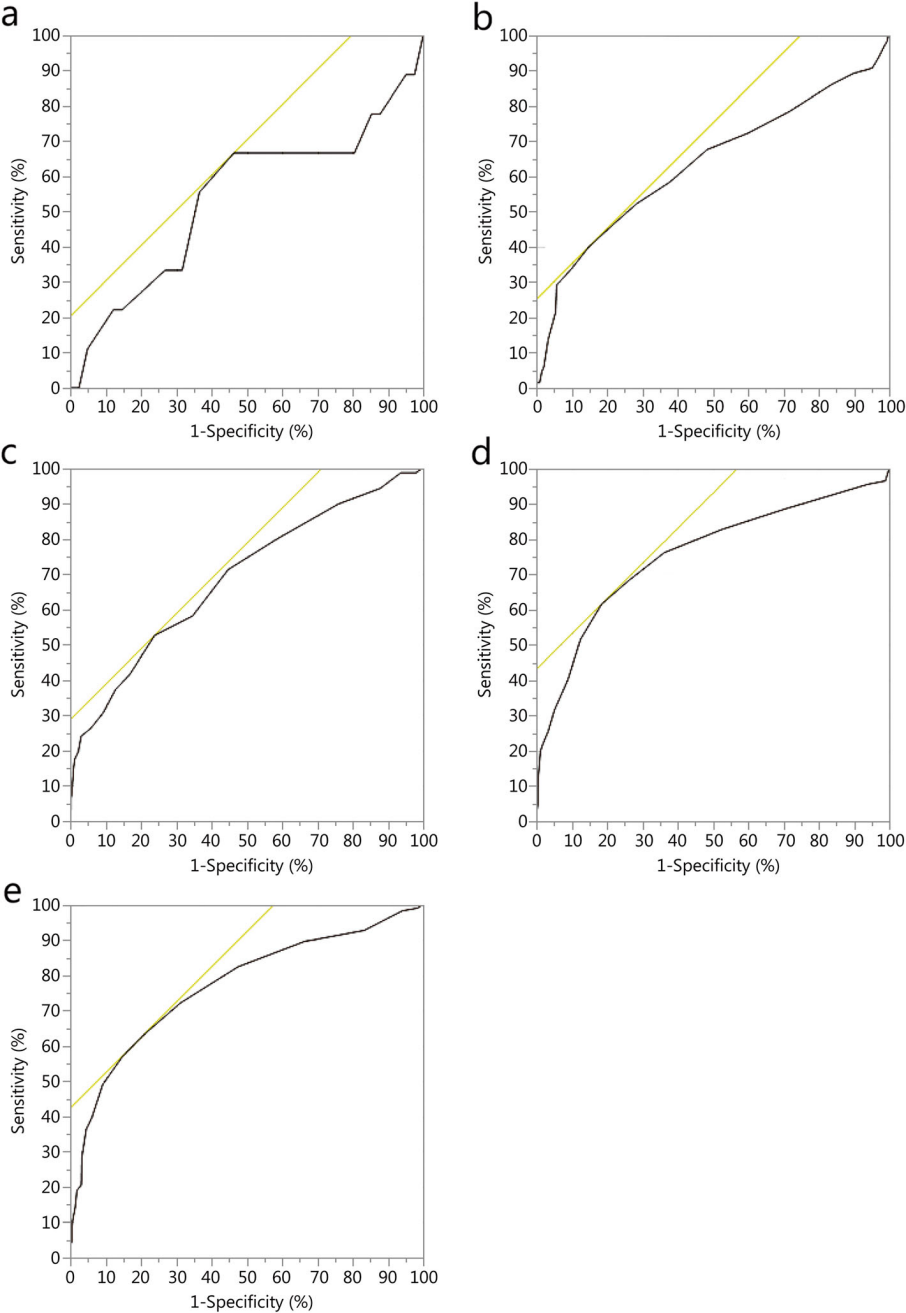
Different age group ROC for predicting massive transfusion. **a**. Age group < 1 year; **b**. Age group 1–3 years; **c**. Age group 4–6 years; **d**. Age group 7–12 years; **e**. Age group 13–17 years.

**Fig. 2:**
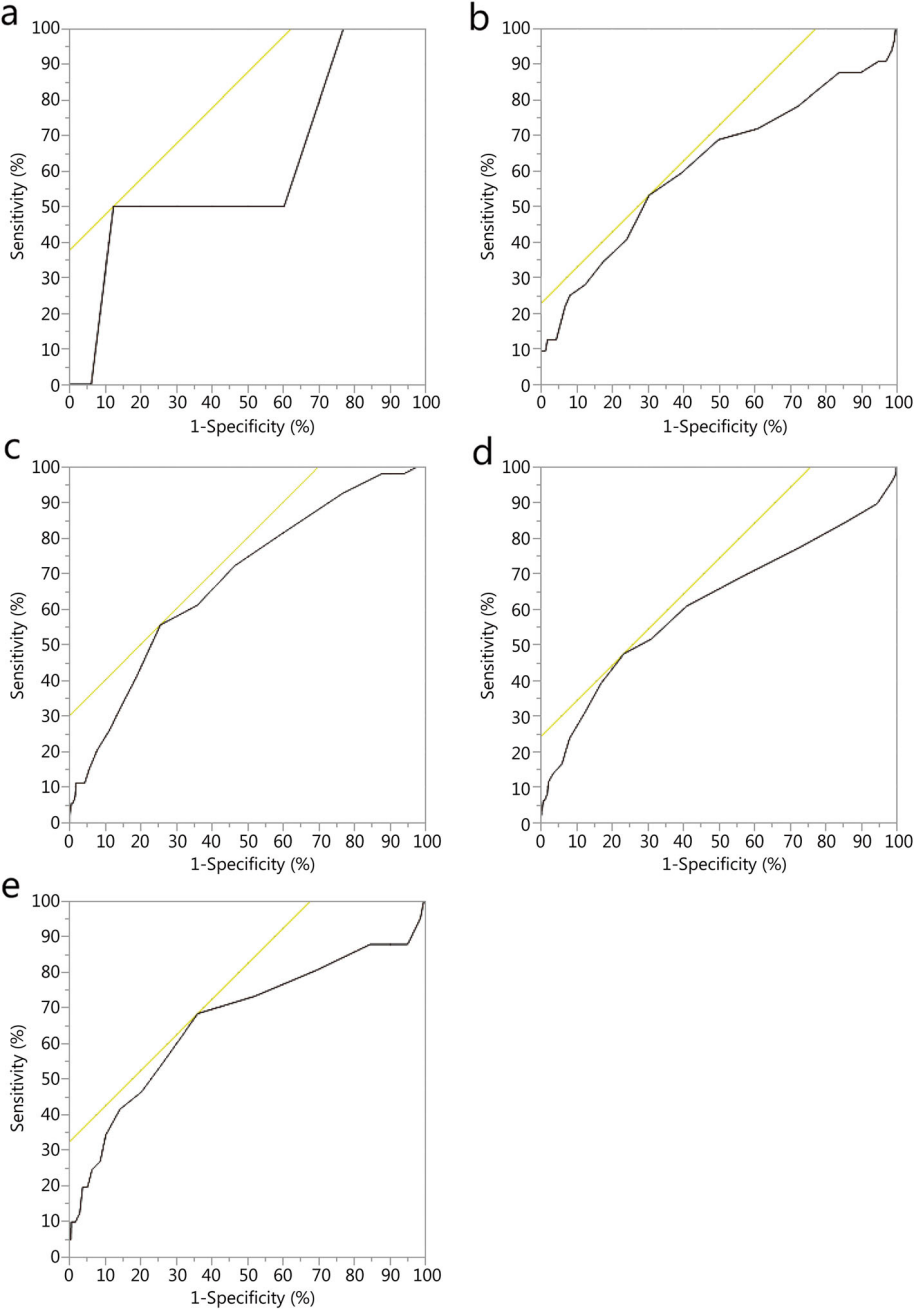
Different age group ROC for predicting death. **a**. Age group < 1 year; **b**. Age group 1–3 years; **c**. Age group 4–6 years; **d**. Age group 7–12 years; **e**. Age group 13–17 years.

From January 2007 through January 2016 there were 42,790 encounters in the DODTR. Of those, 3439 (8.0%) were pediatric by documented or estimated age. Of these, 3145 (91.5%) had both a documented heart rate and systolic blood pressure in the emergency department for inclusion into this analysis. Overall, children that received a massive transfusion (*n* = 502, 16.0%) had a median age of 9 [interquartile range (IQR) 5–13] years, most were male (*n* = 368, 73.3%), injured by explosive (*n* = 301, 60.0%), with serious median injury severity scores (17, IQR 13–25), with most surviving to discharge (*n* = 425, 84.7%). Of the deaths (*n* = 226, 7.2%), the median age was 8 years, most were male (*n* = 160, 70.8%), injured by explosive (*n* = 95, 42.0%), located in Afghanistan (*n* = 149, 65.9%), with serious median injury severity scores (25, IQR 16–29, Table 1). ROC thresholds were inconsistent across age groups ranging from 1.0 to 1.9 with generally limited AUC values for both massive transfusion and mortality prediction characteristics (Table 2, Figs. 1-2). Using the previously defined thresholds for validation, we found overall limited sensitivity and specificity values for both massive transfusion and mortality; however, negative predictive values (NPV) were generally high, with all greater than 0.87 (Table 3).

**Table 1:** Description of casualties based on targeted outcomes

Index	< 1 year (*n* = 50)	1–3 years (*n* = 396)	4–6 years (*n* = 576)	7–12 years (*n* = 1356)	13–17 years (*n* = 767)
Male [*n*(%)]	32 (64.0)	255 (64.4)	399 (69.3)	1094 (80.7)	662 (86.3)
Mechanism of injury [*n*(%)]					
Explosive	14 (28.0)	125 (31.6)	240 (41.7)	655 (48.3)	340 (44.3)
Gunshot wound	6 (12.0)	37 (9.3)	85 (14.8)	303 (22.3)	271 (35.3)
MVC	5 (10.0)	46 (11.6)	91 (15.8)	155 (11.4)	70 (9.1)
Other	25 (50.0)	188 (47.5)	160 (27.8)	243 (17.9)	86 (11.2)
Injury severity score					
Composite [median (IQR)]	8 (1–14)	9 (4–16)	10 (4–17)	10 (5–17)	9 (4–17)
End points [*n*(%)]					
Massive transfusion	9 (18.0)	65 (16.4)	91 (15.8)	211 (15.6)	126 (16.4)
Death	2 (4.0)	32 (8.1)	54 (9.4)	97 (7.2)	41 (5.3)

*MVC* Motor vehicle crash

**Table 2:** SIPA receiver operating characteristic analysis as continuing variable

Variable	ROC threshold	AUC	Sensitivity	Specificity
Massive transfusion				
< 1 year (*n* = 50)	1.4	0.53	0.66	0.54
1–3 years (*n* = 396)	1.8	0.65	0.40	0.86
4–6 years (*n* = 576)	1.4	0.68	0.52	0.77
7–12 years (*n* = 1356)	1.3	0.75	0.61	0.82
13–17 years (*n* = 767)	1.1	0.76	0.62	0.80
Death				
< 1 year	1.9	0.60	0.50	0.88
1–3 years	1.6	0.61	0.53	0.70
4–6 years	1.4	0.68	0.55	0.75
7–12 years	1.3	0.61	0.47	0.77
13–17 years	1.0	0.66	0.68	0.64

*ROC* Receiver operating characteristic; *AUC* Area under the curve

**Table 3:** Analysis of SIPA thresholds ability to predict massive transfusion and death

Variable	Age/Threshold	Sensitivity	Specificity	PPV	NPV
Massive transfusion	1–3 years /SIPA 1.2	0.73	0.35	0.18	0.87
	4–6 years /SIPA 1.2	0.63	0.60	0.23	0.89
	7–12 years /SIPA 1.0	0.80	0.57	0.25	0.94
	13–17 years /SIPA 0.9	0.77	0.62	0.29	0.93
Death	1–3 years /SIPA 1.2	0.75	0.34	0.90	0.94
	4–6 years /SIPA 1.2	0.66	0.59	0.14	0.94
	7–12 years /SIPA 1.0	0.64	0.52	0.09	0.95
	13–17 years /SIPA 0.9	0.70	0.57	0.08	0.97

*SIPA* Shock index pediatric age-adjusted; *PPV* Positive predictive value; *NPV* Negative predictive value

When comparing the AUC for the unadjusted shock index threshold of 0.9 versus the age adjusted, the AUCs were higher for the 1–3 years, 4–6 years, and the 7–12 years groups although this difference was negligible (Table 4). The 13–17 years age group uses the unadjusted SI value, so this is not applicable.

**Table 4:** Side-by-side comparison of the unadjusted shock-index of 0.9 threshold versus the age adjusted thresholds

Variable	Age/Threshold	AUC SI (unadjusted)	AUC SIPA
Massive transfusion	1–3 years /SIPA 1.2	0.50	0.54
	4–6 years /SIPA 1.2	0.55	0.62
	7–12 years /SIPA 1.0	0.62	0.69
Death	1–3 years /SIPA 1.2	0.50	0.54
	4–6 years /SIPA 1.2	0.54	0.62
	7–12 years /SIPA 1.0	0.54	0.58

*AUC* Area under the curve; *SI* Shock index; *SIPA* Shock index pediatric age-adjusted

## Discussion

In this study, we have provided the first description of pediatric trauma cases from within the DODTR to analyze and validate the use of SIPA to predict massive transfusion and mortality in pediatric casualties in Afghanistan and Iraq. Our study expands upon the work of previous reports on the correlation between an elevated SIPA and patient outcomes in blunt trauma. In the case of massive transfusion, SIPA had limited sensitivity and specificity in all age groups. In predicting the outcome of death, SIPA had moderate capability, along with lower sensitivity and specificity. However, the SIPA proved to have a high NPV in all age groups and outcomes, indicating it encompassed most patients in the correct predictive outcomes. While the SIPA had limitations in sensitivity and specificity, the high NPV suggests that it still has some utility and separating out the casualties that were unlikely to require extensive resources. In other words, it will help guide clinicians on when there is less likely a need to active resource-consuming processes such as the walking blood bank.

It is important to acknowledge that the population of pediatric patients observed in this study obtained injuries not typically seen in the civilian setting. SIPA was developed for blunt trauma in the pediatric population and acknowledged a follow would be needed for penetrating trauma [24]. There have been several studies validating the SIPA and its predictive capabilities in civilian hospitals, all expounding on the SIPA’s ability to identify severely injured pediatric patients and accurately differentiate between those with severe and mild injury [14, 15, 24]. The SIPA thresholds outlined by Nordin et al. may not have exhibited the same high predictive capabilities in this study due the drastic difference in injury profiles observed between pediatric cohorts in the civilian and combat environment [5, 15]. The wounds found in a deployed setting, specifically Iraq and Afghanistan are a unique subset of trauma patients in the combat population. While the previous by Nordin and Traynor included penetrating trauma, these are far different than the penetrating trauma seen in the combat setting. Stab wounds are incredibly rare in the combat setting, moreover, the firearms used in combat are frequently higher caliber, higher velocity rounds than seen in the civilian setting. Handguns are the most frequently used firearms in the civilian setting, which comparatively, are rare in the combat setting where long guns are typically employed. The SIPA assessment by Traynor et al. [25] evaluated the SIPA in South Africa – an area with high rates of firearm wounds – found validity, but like the US setting the use of high caliber, high powered rounds is infrequent. Moreover, explosives are not seen in this setting. Pediatric cases with penetrating and blast injuries require further investigations into modifying the SIPA thresholds that would prove valuable for strengthening the SIPA’s predictive capabilities in deployed military settings. Children make up approximately 6% of all admissions in deployed military treatment facilities, therefore, it is important that training and resources for deployed military settings reflect the preparedness that is needed to deal with a high burden of pediatric casualties [7].

There are several limitations of this study. First, we do not have the timeline in which vital signs were documented, so the vital signs may have been documented after interventions were performed. We tried to compensate for this possibility by using the highest documented heart rate and the lowest documented systolic pressure as to not skew the results towards lower SIPA values. Second, for an encounter to be generated within the DODTR, casualties must arrive at a location with surgical capabilities with surgical capabilities, thus we do not know how that may have affected our findings if we included casualties that died prehospital or were pronounced dead upon arrival. Third, our analysis specifically focused on the use of SIPA. Future research that assess the traditional shock index in a pediatric population may be beneficial as having one metric that works for all casualties – adult and pediatric – would simplify the assessments for prehospital personnel. Furthermore, while outside the scope of this analysis, changes in SIPA measured over the course of resuscitation should be assessed as a potential monitoring tool. Other tools have been published, such as the pediatric resuscitation and trauma outcome (PRESTO) model – this may have utility in the combat setting as well [26]. Last, we must rely on the data as documented with the known limitations in deployed data collection which has been previously described [27].

## Conclusions

Within the combat setting, the age-adjusted pediatric shock index had moderate sensitivity and relatively poor specificity for predicting massive transfusion and death. Better scoring systems are needed to predict resource needs prior to arrival, that perhaps include other physiologic metrics. We were unable to validate the previously published findings within the combat trauma population.

## Data Availability

Not applicable.

## Abbreviations

AUC: Area under the curve
DODTR: Department of defense trauma registry
FST: Forward surgical team
ICU: Intensive care unit
ICD-9: International classification of disease 9th edition
JTTR: Joint theater trauma registry
MT: Massive transfusion
NPV: Negative predictive values
PRESTO: Pediatric resuscitation and trauma outcome
ROC: Receiver operating characteristic
SIPA: Shock index, pediatric-age adjusted
